# Promoting Dairy Consumption Among Families: Development and User Experience Study of a Web-Based Nutrition Intervention

**DOI:** 10.2196/66582

**Published:** 2025-08-13

**Authors:** Juliette Lemay, Jacynthe Roberge, Véronique Provencher, Angelo Tremblay, Shirin Panahi, Raphaëlle Jacob, Lucie Brunelle, Gabrielle Saintonge, Vicky Drapeau

**Affiliations:** 1 Quebec Heart and Lung Institute Research Center, Laval University, Quebec, QC, Canada; 2 Institute of Nutrition and Functional Foods, Université Laval, Quebec, QC, Canada; 3 School of Nutrition, Faculty of Agricultural and Food Sciences, Laval University, Quebec, QC, Canada; 4 Institut universitaire de cardiologie et de pneumologie de Québec, Laval University, Quebec, QC, Canada; 5 School of Desing, Laval University, Quebec, QC, Canada; 6 Centre Nutrition, santé et société, Laval University, Quebec, QC, Canada; 7 Department of Kinesiology, Faculty of Medecine, Laval University, Pavillon de l’Éducation Physique et des Sports, 2300, rue de la Terrasse, Quebec, QC, G1V 0A6, Canada, 1 418 656-2131 ext 403874

**Keywords:** IDEAS framework, eHealth, dairy product, family, children, user appreciation, user experience, nutrition, consumption, RCT, randomized, controlled trial, dietary, Canada, behavior change, IDEAS, dairy products, Dairyathlon, ethnographic, interview, planned behavior, questionnaire, diet quality

## Abstract

**Background:**

Insufficient adherence to dietary guidelines underscores the need for effective interventions promoting healthy eating, including dairy consumption, among Canadian families. Research suggests that web-based interventions grounded in user research and behavior change theories can effectively support dietary improvements. However, few theory-driven digital interventions specifically target dairy consumption in families.

**Objective:**

This study aims to describe the development of a web-based nutrition intervention, Dairyathlon, designed to promote dairy consumption among families using the IDEAS (Ideate, Design, Assess, and Share) framework. In addition, it evaluates user experience (UX) with the web-based platform.

**Methods:**

Following the IDEAS framework, family perspectives and beliefs regarding dairy consumption were explored through ethnographic research and interviews. Behavior change techniques, based on the theory of planned behavior, were integrated to enhance attitudes and perceived behavioral control toward dairy intake. These techniques underwent iterative design, prototype testing, and refinement. UX was assessed with the AttrakDiff questionnaire, comparing families using Dairyathlon to those using the Canadian Food Guide (CFG). Children and parents completed the questionnaire after the presentation of the platform (PRE) and following 8 weeks of use (POST). AttrakDiff evaluates pragmatic quality (PQ), hedonic stimulation (HSQ), hedonic identity (HIQ), and attractiveness dimension (ATT) on a scale from –3 to +3, with >1 considered optimal, 0–1 acceptable, and < 0 suboptimal.

**Results:**

Between April 2019 and August 2020, Dairyathlon was developed to enhance families’ attitudes and perceived control over dairy consumption, adhering to the IDEAS framework. Users' experience assessments were conducted among 29 families and showed significantly higher scores for Dairyathlon compared to the reference platform (CFG) at both pre- and postassessments (*P*<.001). Although both platforms were initially rated as optimal, UX ratings decreased after use: PRE (1.7, SD 0.6) to POST (1.4, SD 0.8) in the Dairyathlon group (mean difference of 0.4, 95% CI 0.2-0.7; *P*=.002), and (1.4, SD 0.6) to (0.9, SD 0.6) in the CFG group (mean difference = 0.6, 95% CI 0.5-0.6; *P*<.001). After using Dairyathlon, children (n=45) rated all UX dimensions as optimal, with scores of PQ (1.4, SD 1.0), HSQ (1.6, SD 1.0), HIQ (1.4, SD 1.1), and ATT (1.7, SD 0.9). Parents (n=50) also rated most dimensions as optimal, with scores of 1.2 (SD 1.0) for PQ, 1.4 (SD 0.8) for HIQ, and 1.6 (SD 0.8) for ATT. However, the HSQ dimension received a slightly lower rating of 0.9 (SD 0.8), indicating a need for improvement in adult stimulation.

**Conclusions:**

This study highlights the effectiveness of the IDEAS framework in developing a web-based intervention to promote dairy consumption. The Dairyathlon platform’s UX was rated as optimal, especially for visual attractiveness, though the stimulation dimension requires improvement for adults. Future research will evaluate its impact on dairy consumption, diet quality, and family health status.

## Introduction

Dairy products are known for their specific nutrient content, including protein, calcium, vitamin D, and magnesium, which have well-documented benefits for bone health and growth [1,2]. In addition to high nutrient content, diets rich in dairy products, notably milk, yogurt, and cheese, are known to play a role in the management of body weight and fat composition [3,4]. In one of our studies, an increase in milk consumption (and fruit) was associated with less body weight and fat gain over time [5]. Furthermore, dairy products have a specific food matrix, particularly yogurt, which has been shown to enhance satiety and reduce energy intake in short-term clinical trials [6]. Epidemiological evidence also links regular dairy consumption to reduced risk of chronic diseases [7,8]. According to a study on the Canadian Community Health Survey 2015 cohort, many Canadians do not meet vitamin D, magnesium, and calcium recommendations, all of which are dairy-related nutrients [9]. This portrait reflects the situation before the Canada’s Food Guide (CFG) change in 2019. Since the release of the new CFG, where dairies are no longer presented as a specific category [10], an analysis of nutrient intake provided by a dietary pattern according to the 2019 CFG guidelines suggests a high risk of inadequate intake of calcium and vitamin D [11]. Another study found that the more Canadians adhere to the 2019 CFG, the more they are at risk of calcium and vitamin D deficiencies [12]. Given their significance in growth, nutrient intake, disease prevention, and possibly obesity management, promoting dairy in nutrition programs is relevant, especially since the 2019 CFG no longer distinguishes dairy as a separate group.

Eating habits are acquired early in life. Parents play a pivotal role in teaching healthy eating habits to their children [13]. The family environment, including food availability and parental eating behaviors, influences children’s eating habits [14]. Family-based behavioral interventions have been found effective in fostering the adoption and sustained practice of healthier eating habits [15]. In the context where dairy consumption and healthy eating behaviors are important in children and adults, family intervention should be considered.

Using technology in health promotion is a cost-effective solution that reaches diverse populations [16]. Once implemented, it requires minimal upkeep and can be widely distributed. Grounded in behavior and design theories, web-based interventions offer a promising avenue for promoting healthy family habits [17]. In 2009, our research team developed Team Nutriathlon, a web-based nutrition challenge for schools [18,19], later adapted in 2016 as Family Nutriathlon to encourage fruit, vegetable, and dairy consumption in families [20]. This adaptation successfully increased dairy consumption in the short term, demonstrating the potential of web-based approaches [20]. Building on these findings, this study refines and expands the Family Nutriathlon approach through Dairyathlon, a web-based intervention specifically targeting dairy consumption. Key engagement strategies and family-oriented content from Family Nutriathlon were integrated to enhance Dairyathlon’s effectiveness and sustainability.

To optimize Dairyathlon’s design, we used the Integrate, Design, Assess, and Share (IDEAS) framework, a validated model for developing and adapting web-based health interventions [21]. This structured approach integrates behavior change theory and has been applied successfully in physical activity promotion [22] and health care programs [23]. Given our goal to build on Family Nutriathlon while improving efficacy and usability, IDEAS provided a systematic and efficient framework to guide development. This ensured that Dairyathlon was tailored to family needs while maximizing engagement and impact.

This study aims to describe the development process of a web-based nutrition intervention to promote dairy products consumption among families using the IDEAS framework and to evaluate users’ appreciation of the web-based platform compared to a reference nutrition platform. The hypotheses are: (1) designing the platform based on the IDEAS theoretical framework will lead to a web-based platform that users highly appreciate and (2) both children and parents are expected to find the platform more enjoyable and useful than the reference nutrition platform, as it is specifically designed to address the needs of Quebec families.

## Methods

### Overview

**Figure 1.**
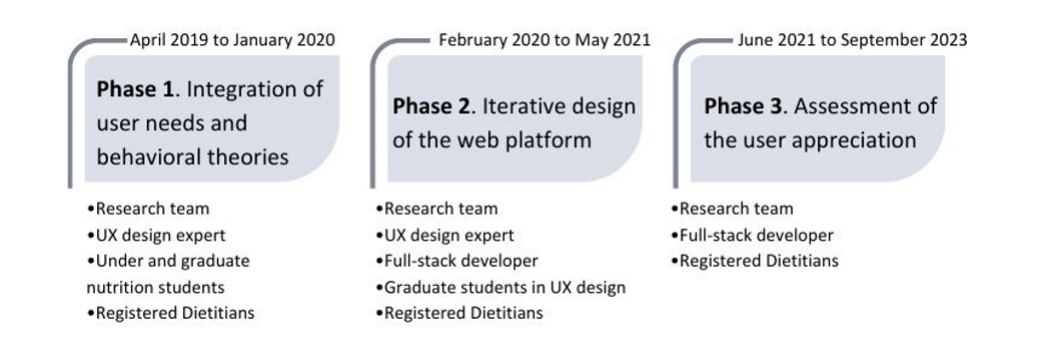
Overview of the Dairyathlon development process: timeline, phases, and human resources. UX: user experience.

This study adapted the Family Nutriathlon web-based intervention [20], developed to promote healthy eating among families. It was selected for adaptation because of its beneficial effect on dairy intakes in the short term, its similar targeted users, the inclusion of health behavior change techniques (ie, self-determination theory), and its focus on specific food groups such as dairy consumption. The adaptation process included 3 phases inspired by the IDEAS framework. In phase 1, the focus was on integrating the target users and specifying the target behavior. Phase 2 involves the design process in an iterative process (ie, designing the user interface and experience and creating features of the platform). Phase 3 assessed the user experience (UX) of the platform’s design (see Figure 1).

### Phase 1: Integration of User Needs and Behavioral Theories

Phase 1 of the IDEAS framework sought to gather insights from the target users, specify the target behavior, and ground in behavioral theory [21]. As specified by Curtis et al [24], the target population should be included in all steps of the development process. In this study, our target population included families with children aged between 8 and 16 years. The age of 8 years marks the minimum age at which children can reliably complete questionnaires and navigate on the web platform, while 16 years is the typical upper limit for parental influence on food choices. In addition, this age range aligns with calcium needs during growth and was effective in our previous Nutriathlon study [20]. To better understand the targeted population’s thoughts, opinions, and experiences, an ethnographic process was undertaken [25]. First, a 27-item questionnaire was created to explore families’ activities, dairy consumption, grocery shopping habits, cooking, meal planning, time availability, perception of clinical studies, and use of electronic devices. This questionnaire was shared on Facebook (Meta Platforms) groups targeting parents living in Quebec, Canada. The questionnaire was designed by the UX design team in collaboration with the nutritionists from the research team. The questions were not validated before being sent to the target population. The survey was voluntary and open to all site visitors, but only participants with at least one child between 8 and 16 years old were retained for analysis. No incentives were offered. In May 2019, for 1 week, a total of 327 families completed the internet-based survey. Second, to complement the quantitative results, we conducted 7 interviews among the families who agreed to be contacted, to explore their needs, eating habits, and motivations toward dairy consumption. Based on the results from the questionnaire and the interviews, two family profiles were established: (1) A young family with children (8‐12 y): they cook on weekends, plan meals, maintain a well-stocked kitchen, and aim to expand their nutrition knowledge. They consume a lot of milk and moderately consume yogurt and cheese. (2) A family with teenagers (13‐16 y): teens participate in meal preparation and grocery, meal choices vary among family members, and their motivation is driven by competition and rewards. They prefer cheese and consume less yogurt and milk. These profiles were considered during the next steps (ie, the design process) to ensure that the intervention is specifically created for the target user and that it is based on the family’s needs.

Second, we specified the target behavior. Since we aimed to develop a web-based nutrition intervention to increase dairy consumption, we defined the target behavior as consuming 4 dairy servings per day (ie, 250 ml of milk, 50 g of cheese, and 2 servings of 175 ml of yogurt). This quantity provides 1200 mg of calcium, which has been shown to improve appetite and body weight control in adults and children [26]. All flavored types, including sweetened and plain, were permitted to enhance yogurt acceptability. Since the family interviews revealed that they were less structured and usually overloaded on weekends, only weekdays (ie, Monday through Friday) were targeted for the intervention to accommodate families.

Finally, the next step was to ground in behavior theories. The theory of planned behavior (TPB), which has emerged as particularly effective in web intervention on healthy lifestyle habits aimed at changing behavior [27], was used to target 2 determinants of behavioral change: attitude and perceived behavioral control toward dairy consumption. From each determinant, salient attitude beliefs (ie, advantages and disadvantages of consuming dairies), and salient control beliefs (ie, facilitators and barriers to dairy consumption) were assessed [28]. First, parent’s salient beliefs related to dairy consumption were identified based on a qualitative study from Lacroix et al [29], where beliefs about dairy products were gathered during focus group sessions among 161 men and women aged 16 to 50 years in the Quebec City, Montreal, and Toronto regions. Second, in the absence of existing literature to identify salient beliefs in children, our research team developed a questionnaire based on the key elements from the study by Lacroix et al [29]. In December 2019, the 18-item questionnaire was sent by email to a family mailing list from Université Laval. Parents who received the questionnaire were informed of its objective: to identify the key beliefs related to dairy consumption among children aged 8 to 16 years. The first question asked about the child’s age, and only responses from children within this age range were accepted. No financial compensation was provided. A total of 87 children in the Quebec City area completed the questionnaire. Attitude and perceived control about dairy consumption were then targeted with a specific behavior change technique (BCT). These BCTs were selected from the taxonomy by Michie et al [30], such as pros and cons, health consequences, instruction on how to perform a behavior, and social comparison, to favor a positive attitude and increase perceived behavioral control toward dairy consumption. In addition, motivational aspects, inspired by Robinson [31], were incorporated, such as gamification [32], goals and visual representations of the goals [33], a leaderboard, and digital trophies (eg, badges displayed on the intervention platform) [25]. A challenge between families was added in the web-based intervention, where families could earn points and symbolic prizes by meeting dairy consumption targets or trying some of the BCTs available on the web-based platform, such as true or false quizzes, tips and advice section, and recipes. The leaderboard, one important gamification feature of the intervention, recognized high-achieving families anonymously, promoting healthy competition and motivation [25]. The salient beliefs linked to the selected BCTs are outlined in Multimedia Appendix 1.

### Phase 2: Iterative Design of the Web Platform

After identifying the BCTs for the features of the Dairyathlon platform, a collaborative effort involving nutrition researchers, the UX design team, full-stack developer, and the registered dietitian team was undertaken in an iterative design process. Phase 2 involved a human-centered design process focusing on nutrition challenge and education content, the conceptualization of the platform, and usability tests.

Initially, the education information content, based on the salient beliefs identified in the first phase, was developed and validated by a team of dietitians, ensuring that the literacy level was appropriate for children aged 8 to 16 years. An informal review of the content was conducted with a group of 3 children and 3 parents, who provided qualitative feedback on clarity of the content. Based on this feedback, adjustments were made to improve understanding.

The UX design team prototyped the web-based intervention, starting with wireframes to visualize the platform. After refining these with the research team, flowcharts were created to map out user navigation. With the help of a full-stack developer, the first versions of the intervention were developed for both mobile and computer formats.

A total of 2 rounds of usability testing were conducted to refine the platform. The A/B test compared 2 versions of the intervention, asking users to indicate their preferences (eg, whether a data table with numerical values or color-coding was more effective). The A/B test involved observing users performing tasks on the platform, guided by an audio scenario, and evaluating success, partial success, or failure for each task. Feedback from the tests, including task completion time and user satisfaction, helped identify areas for improvement.

Only 1 round of testing was made, involving 5 participants (3 adults and 2 children), and adjustments were made based on feedback, which was key in improving usability. The final version of the Dairyathlon platform was developed in collaboration with a full-stack developer, and project management tools (eg, DevOps and Microsoft Azure) ensured efficient tracking of the project’s timeline and features. This platform was designed to be highly flexible and easily adaptable. For example, it allows for the addition of new food groups or modifications to better accommodate different target audiences and populations. The platform is hosted on Université Laval’s secure servers, allowing for the simultaneous registration of multiple families. It is accessible across computers, tablets, and mobile phones. Security measures include password authentication, SSL encryption, and real-time access monitoring. The technical architecture and security of the platform are summarized in Multimedia Appendix 2.

### Phase 3: Users Experience Assessment

The third phase of the study focused on assessing the UX of the Dairyathlon platform among families. This assessment was carried out using the AttrakDiff questionnaire before and after an 8-week intervention period. Following sections present the UX assessment methodology.

### Ethical Considerations

This study was approved by the Research Ethics Board of Université Laval (approval NCT05417347). All parents and children provided written informed consent and assent, respectively, which explicitly included approval for analyses of the collected data (AttrakDiff). As part of the informed consent process, participants were also informed about data privacy and confidentiality measures. All research documents will be stored in a coded (deidentified) form for 5 years and then anonymized and retained for 25 years by the principal investigators. Participants in the intervention group received free yogurt coupons as compensation for the cost of the dairy products. No monetary compensation was offered to participating families.

### Participants

Families were recruited between June 2021 and September 2023 in the Quebec City area through various channels, including Facebook family groups, email lists from Université Laval and primary schools, and public spaces like community centers. This study was single-blind, meaning that participants were unaware of whether they had been randomized to the intervention or control group. Recruitment materials described the study’s objective as evaluating a family-based nutrition intervention, without specific reference to dairy products. Interested families could contact the research team via email or phone for further information and to complete an eligibility screening. The inclusion criteria were parents with children aged 8 to 16 years, with at least one parent presenting overweight (BMI ≥27 kg/m² and a waist circumference ≥88 cm for females and ≥102 cm for males). Families with food allergies or dairy restrictions were excluded.

### Intervention and Control Group

Families were randomly assigned to intervention or control group for the 8-week intervention. The intervention group received a 1-hour face-to-face presentation of the Dairyathlon challenge, which involved the consumption of 4 dairy servings/day and was introduced to the Dairyathlon web-based platform. They were encouraged to use the Dairyathlon web-based platform during the 8 weeks to help them reach their dairy consumption target and participate in the challenge as a family. The control group received a 1-hour presentation on the 2019 CFG website, which provides dietary recommendations, including the healthy plate model (half fruits and vegetables, one-quarter protein, and one-quarter whole grains) [10]. They were invited to consult the website and follow its recommendations for the 8-week period. During the 8-week intervention, all families had 4 nutritional follow-up sessions with a dietitian every 2 weeks. The dietitian checked progress toward goals and monitored the use of the Dairyathlon platform. The families’ attendance at the follow-up meetings allowed us to measure the fidelity of platform use.

### Measurement

UX was measured before (PRE) and after (POST) the 8-week intervention using the validated French version of the AttrakDiff questionnaire for parents and a validated short version for children [34-36]. The AttrakDiff questionnaire, developed by Hassenzahl et al [36], is designed to assess the UX of interactive products. It consists of 28 pairs of opposite items (eg, “good – bad”) on a 7-point Likert scale for adults and 10 pairs for children. The questionnaire evaluates four dimensions: (1) Pragmatic quality (PQ): practicality and functionality of the platform, (2) Hedonic-stimulation quality (HSQ): joy of use and the sense of challenge, (3) Hedonic-identity quality (HIQ): Personal expression and control of one’s image in a social context, and (4) Attractiveness dimension (ATT): visual appeal of the interface. Each dimension is scored from −3 (very negative) to +3 (very positive). Scores below 0 are suboptimal, scores between 0 and 1 are acceptable, and scores above 1 are optimal [36]. A global score is calculated, representing the overall UX appreciation [37]. This evaluation allows a comparison of the Dairyathlon platform to a reference platform (in this case, the CFG website). The AttrakDiff questionnaire provides insight into the strengths and weaknesses of the Dairyathlon platform, with a specific focus on differences in UX between children and parents.

### Statistical Analysis

Participants were randomly assigned to the intervention or control group using a computer-generated randomization list. The randomization was performed before the start of the intervention to ensure unbiased assignment. As the statistical power required for evaluating user appreciation with the AttrakDiff questionnaire was achieved (ie, more than 20 participants), these analyses were conducted [38]. Statistical analyses were performed only in families who completed the full 8-week intervention. For the global appreciation assessment of both Dairyathlon and CFG platforms, paired *t* tests and Welch *t* tests (when assumptions were not met) were used to assess changes over time within groups (PRE vs POST in each group). An independent *t* test was conducted to compare the Dairyathlon group with the control group (CFG) at both PRE and POST. Only families who completed the full 8-week intervention were included in the analysis.

The AttrakDiff questionnaire (UX) used paired *t* tests to compare pre- and post-ratings for each dimension, assessing significant changes over time in children and parents. This analysis focused exclusively on children and parents in the Dairyathlon intervention group, as the primary goal was to assess the UX design of the platform. Data are presented as mean (SD), with statistical significance set at *P*<.05. Statistical analyses were performed using IBM SPSS 29.

## Results

### Visual of the Dairyathlon Platform

**Figure 2.**
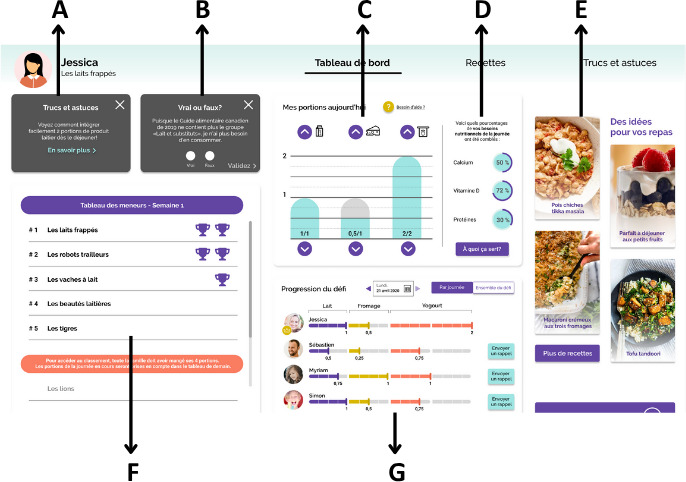
Dairyathlon homepage design (Dashboard), computer format. (A) Tips and advice section; (B) True or false quizzes; (C) Compilation tool; (D) Daily value in %; (E) Recipes; (F) Leaderboard; (G) Challenge progression view with family member avatar.

**Figure 3.**
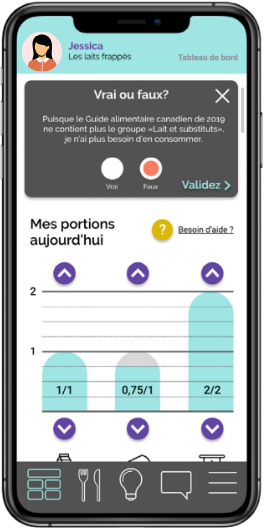
Mobile format of the Dairyathlon web-based intervention with true or false quizzes and the compilation tool.

In May 2021, the final version of the Dairyathlon platform was launched via the Université Laval server, ready to be used by the families. Figure 2 illustrates the visual of the web-based platform, and Figure 3 shows the mobile representation.

### User Experience

#### Global Appreciation

As of January 2024, a total of 44 families completed the full 8-week intervention. In the intervention group, 29 families were randomized, and 15 families were randomized in the control group. The study is expected to conclude in February 2025, with a target of 60 families. In the intervention group, 42 children and 46 parents completed the AttrakDiff questionnaire before and after the intervention, while 26 children and 28 parents in the control group. Platform usage compliance was assessed based on attendance at follow-up sessions with the dietician. Families in the intervention group attended an average of 3.5 out of 4 sessions (88%), while families in the control group attended an average of 3.8 out of 4 sessions (95%).

**Figure 4.**
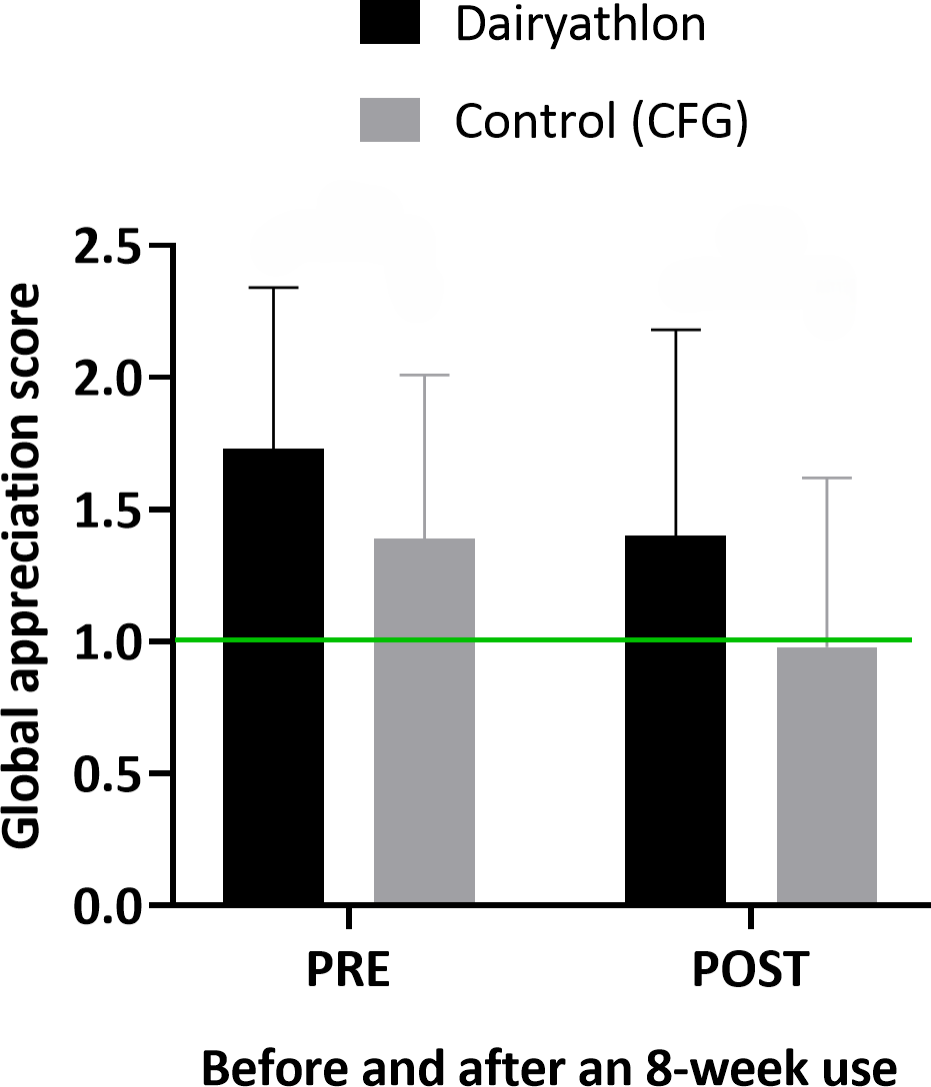
Family global appreciation score, before (PRE) and after (POST) an 8-week use of the Dairyathlon platform and the nutrition reference platform (Canada’s Food Guide). Significant differences were observed before (PRE) *P*<.001, and after (POST) *P*<.001, and the green line indicates the minimal score for optimal. CFG: Canada’s Food Guide

First, results show a significant decrease in scores from PRE to POST in both groups. In the Dairyathlon group, the mean score decreased from 1.7 (SD 0.6) at PRE to 1.4 (SD 0.8) at POST, with a mean difference of 0.4 (95% CI 0.2-0.7), significant at *P*=.002 (paired *t* test, assuming equal variances). In the control group (CFG), the mean score dropped from 1.4 (SD 0.6) to 0.9 (SD 0.6), with a mean difference of 0.6 (95% CI 0.5-0.6), significant at *P*<.001 (Welch-Satterthwaite *t* test, due to unequal variances). An independent *t* test revealed a significant difference between the two groups at both time points, with the Dairyathlon group scoring higher than the control group at both PRE (*P*<.001) and POST (*P*<.001). Figure 4 illustrates these results.

#### Dimensions Appreciation

**Figure 5.**
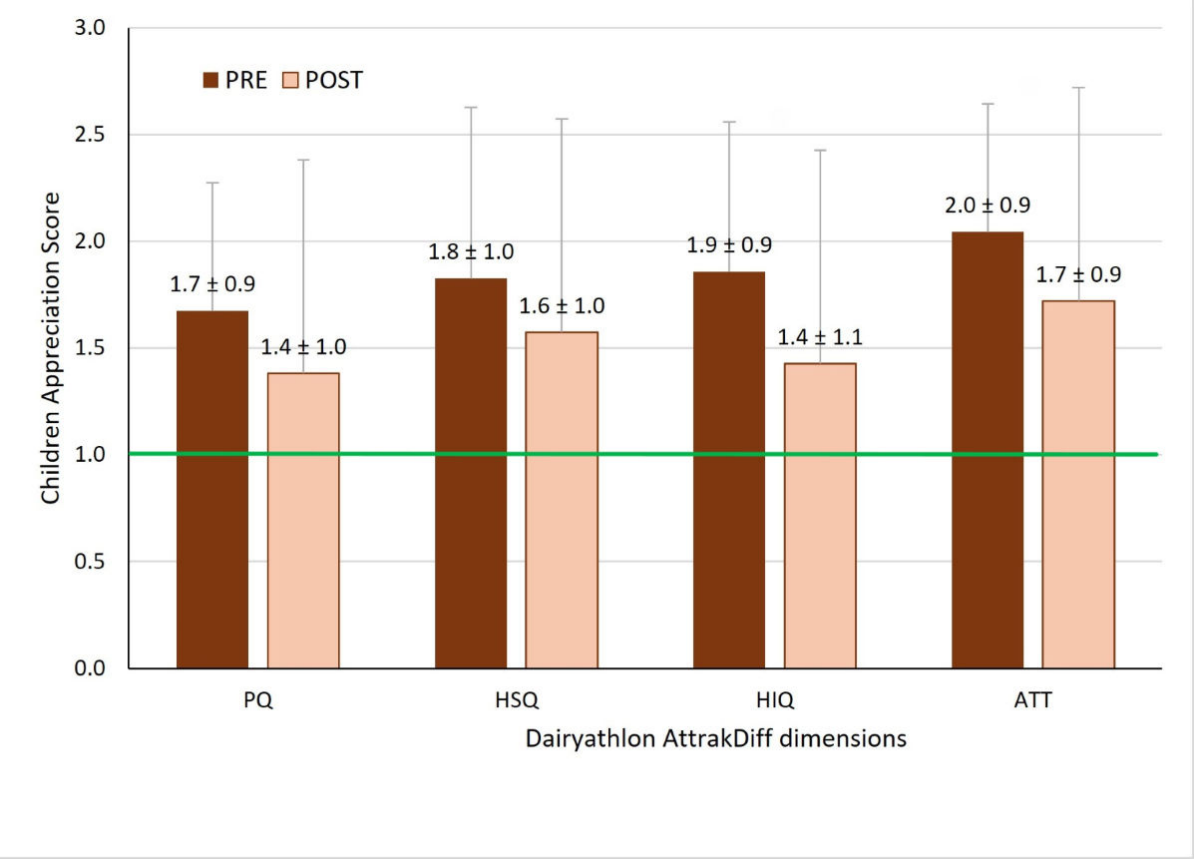
Children's user experience of the Dairyathlon platform, before (PRE) and after (POST) an 8-week use for pragmatic quality (PQ), hedonic-stimulation quality (HSQ), hedonic-identification quality (HIQ), and attractiveness dimension (ATT). *P*<.05 for HIQ and ATT. The green line indicates the minimal score for optimal.

**Figure 6.**
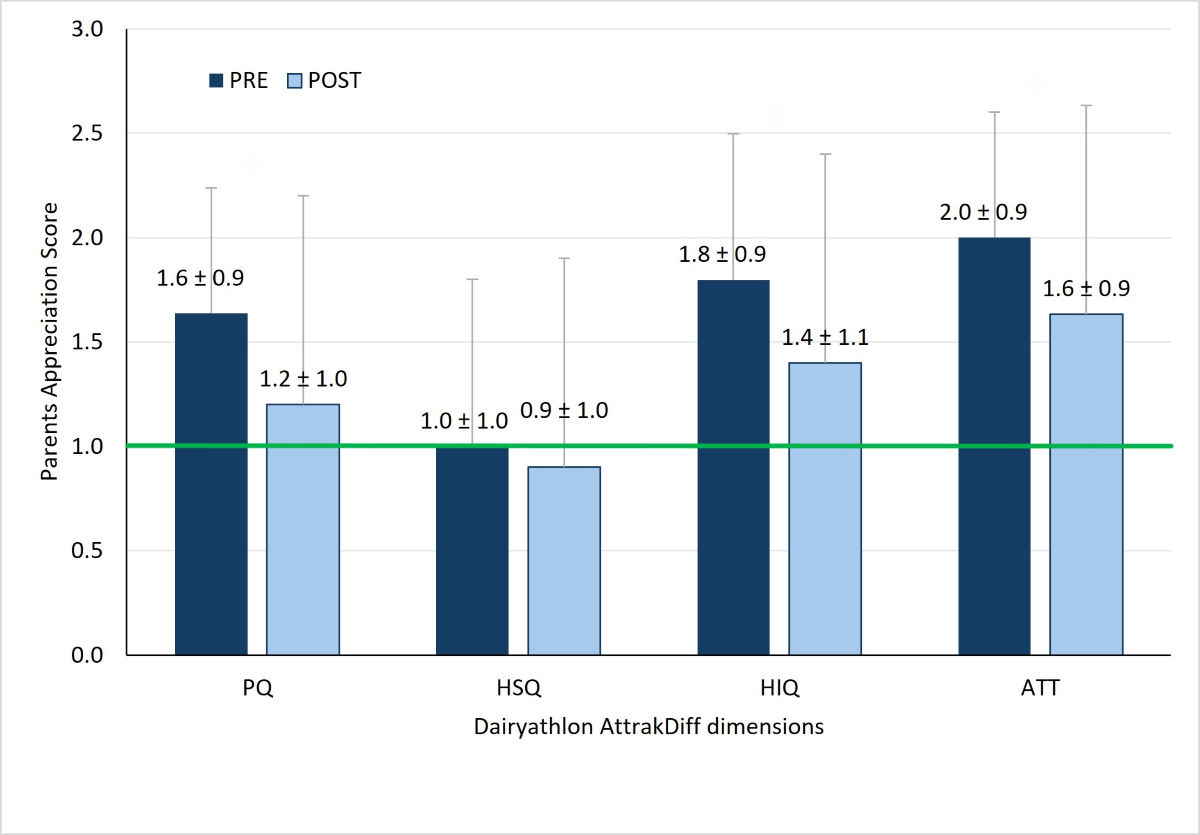
Parents' user experience of the Dairyathlon platform, before (PRE) and after (POST) an 8-week use for pragmatic quality (PQ), hedonic-stimulation quality (HSQ), hedonic-identification (HIQ) qualities, and attractiveness dimension (ATT). *P*<.05 for PQ, HIQ, and ATT. The green line indicates the minimal score for optimal.

In the 29 intervention families, 42 children and 46 parents completed the AttrakDiff questionnaire before and after the 8-week intervention. Among children, all 4 dimensions were rated above 1 at both time points (see Figure 5) , while parents only rated all the dimensions above 1 before the intervention (see Figure 6). After the intervention, parents rated the stimulation dimension as acceptable. For both children and parents, the highest dimension before and after the use of the Dairyathlon platform is related to its aesthetics (attractiveness).

Paired *t* tests were conducted to compare PRE and POST ratings for each dimension to determine if scores significantly decreased over time. For children, the HIQ (1.7, SD 0.9 vs 1.5, SD 0.8; *P*=.04) and ATT (2.0, SD 0.9 vs 1.7, SD 0.9; *P*=.01) dimensions showed significant decreases (see Figure 5).

For parents, significant decreases were observed in the PQ (1.6, SD 0.9 vs 1.2, SD 1.0; *P*=.004), HIQ (1.8, SD 0.9 vs 1.4, SD 1.1; *P*=.001), and ATT (2.0, SD 0.9 vs 1.6, SD 0.9; *P*<.001) dimensions (see Figure 6).

**Figure 7.**
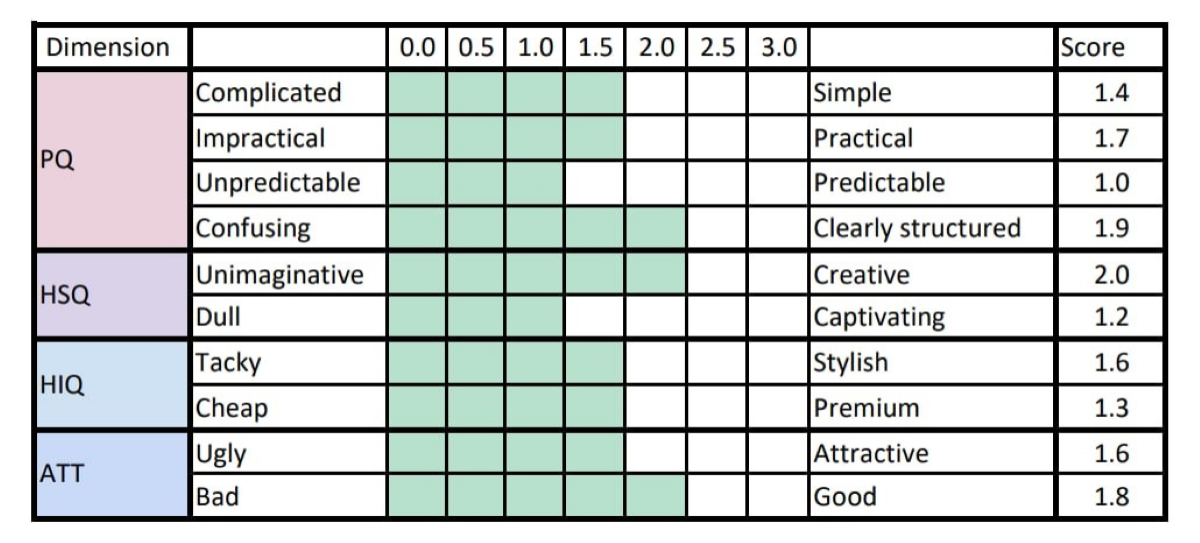
Children's user experience score for each item (10), after its use. Dimensions: pragmatic quality (PQ), hedonic-stimulation quality (HSQ), hedonic-identity quality (HIQ), and attractiveness dimension (ATT).

After its use, items with the highest score for children were: “Clearly structured,” “Creative,” and “Good,” while the lowest scores were “Unpredictable” and “Dull” (see Figure 7).

**Figure 8.**
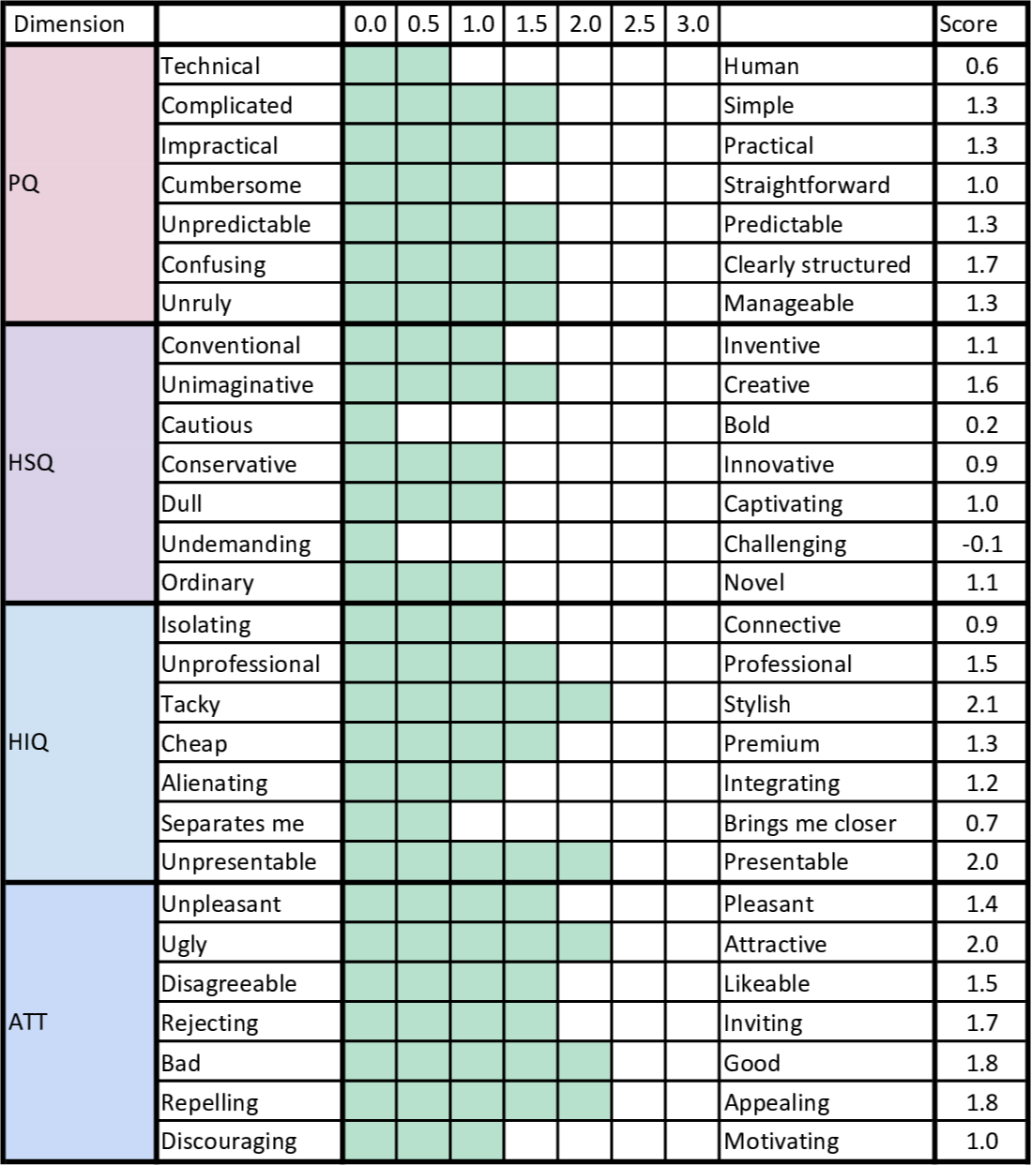
Parents’ user experience score for each item (28), after its use. Dimensions: pragmatic quality (PQ), hedonic-stimulation quality (HSQ), hedonic-identity quality (HIQ), and attractiveness dimension (ATT).

For parents, the lowest items were: “Cautious” and “Undemanding,” suggesting that the gamification part (hedonic-stimulation dimension) was not optimal to stimulate them (see Figure 8).

## Discussion

### Principal Findings

This study examined the development process and UX of Dairyathlon, a web-based nutrition intervention designed to promote dairy consumption among families. Guided by the IDEAS framework, the development process began with assessing families’ needs and beliefs about dairy consumption. An iterative, multidisciplinary approach integrated gamification and educational elements to enhance engagement. The Dairyathlon platform provided an optimal UX, outperforming the reference nutrition website both before and after use. While children responded positively to the platform, adults showed lower appreciation, particularly in the stimulation dimension, underscoring the challenge of designing universally appealing family-based interventions. Despite differences in engagement levels among users, the Dairyathlon shows promising potential for improving dietary habits in families. Beyond its immediate impact, investing in the platform could contribute to long-term benefits, including better bone health, a reduced risk of chronic diseases, and enhanced nutrition literacy. This study is part of an ongoing randomized controlled trial, and future studies will assess the short-term (after an 8-week use) and long-term (4 months after) effects of the Dairyathlon platform on dairy consumption, diet quality, and weight management among families.

Grounding the intervention content in behavioral theory, in the present case the TPB, was a complex and long process due to the lack of studies in the literature on dairy consumption beliefs. In this study, a qualitative analysis was conducted on adults’ beliefs about dairy, but research on children was still required. The identification of dairy consumption salient beliefs was important for targeting BCTs aimed at fostering positive attitude and decreasing barriers toward the consumption of dairy products [39]. Recognizing the salient belief in adults and children was key to identifying specific BCTs and the platform features. Supporting this approach, a meta-analysis by Webb et al [27] revealed a correlation between the use of BCTs and the effectiveness of web-based interventions. This further underscores the importance of a well-founded theoretical basis in designing impactful health promotion strategies.

The IDEAS framework ensures high-quality scientific communication and supports the development of interactive user-centered content [25]. The iterative design process within a multidisciplinary team helps create evidence-based and targeted content for users, though it may extend development timelines [40]. Despite initial and ongoing costs for software, security, and content maintenance, web-based platforms like Dairyathlon offer economic advantages over traditional interventions by reducing individual counseling time, travel, and printed materials. While few studies have applied the IDEAS framework to web-based behavior change interventions, examples in physical activity and nutrition suggest its relevance [22,41]. In a randomized controlled trial, a mobile app designed with this framework led to increased vegetable consumption among overweight adults [42]. These applications highlight the framework’s adaptability for health-related behavioral interventions.

The global UX assessment using the AttrakDiff questionnaire showed that families were generally satisfied with the Dairyathlon platform. When compared to the reference nutrition platform, the CFG, the global appreciation for the Dairyathlon platform was higher before and after its use, highlighting the importance of a UX design approach in the development of web-based nutrition interventions. However, both the Dairyathlon and the reference platforms showed a decrease for user appreciation over time, which can suggest a familiarity effect [43,44]. A decline in user engagement with interactive web-based platforms appears to be common, particularly when gamified elements are involved, as they can lead to boredom if the content becomes too simplistic for sustained interest [45,46]. While long-term studies on web-based nutrition platforms are limited, research on other eHealth interventions has shown a decrease in user interest after 3 to 6 months of use [47]. To encourage longer-term engagement, future iterations of the platform could include weekly new interactive quizzes, complemented by email notifications to prompt ongoing participation, as outlined in a recent scoping review [48]. Furthermore, features such as push notifications for new recipes or challenges and a chat system with a registered dietitian could promote more regular engagement. However, time and funding constraints limited the development of these features in this study.

The assessment of each dimension before and after using the Dairyathlon platform helped identify both the strengths and weaknesses of the platform. Although evaluating UX with AttrakDiff is suggested before and after use [36], most studies assess it only at baseline [49].

First, for children, the ratings were optimal across all dimensions, which is promising. A study assessing the relationship between children’s UX satisfaction and learning in a nutrition-focused video game found that UX satisfaction was positively correlated with their learning outcomes among children aged 8 to 10 years [50]. However, these results should be interpreted with caution, as validation studies of the AttrakDiff short version among children are still limited. To our knowledge, only a single study has assessed the internal consistency of the AttrakDiff dimensions in children, finding no significant associations [35], and another study has evaluated the AttrakDiff short version (10 items) among adults, reporting internal consistency [51]. More studies are needed to validate the use of the AttrakDiff short version in children further. While future research will assess the impact of the Dairyathlon intervention on dairy consumption, this study provides promising results, particularly in terms of children’s satisfaction and potential learning outcomes. For parents, among the 4 UX dimensions, only hedonic stimulation was suboptimal, indicating that the gamification elements did not provide enough challenge or engagement. This suggests that the tasks and rewards may have been too simplistic or not well aligned with the parents’ needs and motivations. In our study, we observed that parents tended to complete the quizzes early on, often within the first few weeks. To increase adult engagement, future interventions could introduce more structure and interactive tasks with additional educational content. Weekly quizzes increasing in difficulty levels could be introduced, with email notifications, which have been shown to increase dairy-derived calcium intake and could inform parents about new quizzes and content to boost participation [52]. Tailored features, for example, educational videos with nutrition tips or interactive quizzes for parents and children, could help maintain attention, engagement, and support learning [48,53].

### Limitations

Our study had several limitations. The sample was limited to participants from the Quebec region, Canada, which may affect the generalizability of the results. The platform could be adapted for other cultural contexts using the IDEAS framework. This would first require an assessment of the target population’s needs, cultural beliefs, and attitudes toward dairy consumption. Following this, both the content and the format would need to be adjusted to align as closely as possible with the target culture. As highlighted in a recent narrative review, ensuring a culturally relevant and accessible UX, through appropriate design and translation, is essential for effective adaptation [54]. Another limitation of this study is that it relied solely on quantitative evaluations of UX, which may limit the depth of result interpretation. A qualitative study involving interviews with families who used the Dairyathlon platform would have provided valuable insights beyond the AttrakDiff score, highlighting specific features that were particularly appreciated. If the platform is adapted for broader use in the future, conducting qualitative studies with user families and organizing focus groups would be important for refining and enhancing the platform. Finally, this study did not cover all steps of the IDEAS framework, including evaluating our web-based intervention’s effectiveness and impact on dairy consumption among families [21]. These last steps are part of another ongoing study by this study’s present team.

### Conclusion

This study outlines the development process of a web-based nutrition intervention for families using the IDEAS framework. It includes a concrete example and discusses timelines, issues, and reflections. The UX assessment of the Dairyathlon platform, using AttrakDiff, showed optimal appreciation before and after its use, surpassed the reference nutrition platform, and stimulation dimension for parents could be improved.

## Supplementary material

10.2196/66582 Multimedia Appendix 1 Behavior change techniques (BCTs) targeting adults’ and children's salient beliefs and motivation toward dairy consumption.

10.2196/66582 Multimedia Appendix 2 Overview of platform architecture and security features.

## Abbreviation:

ATT: attractiveness dimension
BCT: behavior change technique
CFG: Canada’s Food Guide
HIQ: hedonic-identity quality
HSQ: hedonic-stimulation quality
IDEAS: Ideate, Design, Assess, and Share
PQ: pragmatic quality
TPB: theory of planned behavior
UX: user experience
