# Supplementary material for: Promoting Dairy Consumption Among Families: Development and User Experience Study of a Web-Based Nutrition Intervention
Source: JMIR Form Res. 2025 Aug 13;9:e66582. doi: 10.2196/66582 (PMC12349888; doi:10.2196/66582)
Supplement: Multimedia Appendix 1 [file formative-v9-e66582-s001.docx]

Table 1. Behavior change techniques (BCTs) targeting adults’ and children's salient beliefs and motivation toward dairy consumption.

| Behavioral determinants | Salient beliefs | BCT from Michie et al.^a^ | Features of the web-based platform |
| --- | --- | --- | --- |
| Attitude | **Advantages:**   Health benefits, nutritional advantages, Nutritiousness, naturalness, and taste (A+C)   Convenience, socioeconomic impacts (A)   Easy and quick to consume (C)   **Disadvantages:**   Organoleptic properties and processed products (A+C)   Adverse health effects, nutritional disadvantage, inconvenience, values (origin), and guilt (A)   Better when fresh (C) | Persuasive argument [9.1]    Pros and cons [9.2]    Health consequences [5.1]    Social and environmental consequences [5.3]    Feedback on behavior [2.2] | **True or false quizzes**: Explain the benefits of dairy consumption and demystify false beliefs.     **Information about daily value in %**: Improve knowledge about dairy products by showing protein, calcium, and vitamin D intakes reached with dairy consumption. |
| Perceived behavioral control | **Facilitators were the opposite of the following barriers**:   Limited access, lack of cooking inspiration, difficult lunch box storage (A+C)   High price, health status, reduced confidence, supply-side issues, cultural habits and values, social influences,  and unpleasant smell (A) | Instruction on how to perform a behavior [4.1]    Problem solving/coping planning [1.2] | **Recipes**: Increase perceived control with recipe pictures, ingredients, and time preparation.     **Tips and advice section**: Instruction on how to perform a behavior, to decrease barriers and increase facilitators (e.g., How to keep milk fresh in the lunch box). |
| Motivation | Try new recipes (A+C)   Family involvement and nutritional advice (A)   Games, challenges, and rewards (C) | Social support (practical) [3.2]    Goal setting (behavior) [1.1]    Self-monitoring of behavior [2.3]    Prompts/cues [7.1]    Social comparison [6.2]    Modelling of the behavior [6.1]    Review behavior goal(s) [1.5]    Non-specific reward [10.3] | **Compilation tool**: Documenting dairy consumption of each family member to increase users’ engagement.  **Team family challenge**: Participation of the whole family.  **Leaderboard:** Visual ranking between families according to the achievement of objectives.  **Reminder button**: Available to the family leader of the challenge.  **Virtual trophies**: Earned when dairy products are consumed or BCTs are used (e.g.: Try a recipe). |

Table Legend: Salient beliefs of adults and children were combined in one column due to similarities: (A) adults and (C) children.

(a) BCTs are coded according to the Behavior Change Techniques taxonomy from Michie et al. (2013). The numbers in brackets correspond to the standardized BCT identifiers from this taxonomy [30].
