# Supplementary material for: Promoting Dairy Consumption Among Families: Development and User Experience Study of a Web-Based Nutrition Intervention
Source: JMIR Form Res. 2025 Aug 13;9:e66582. doi: 10.2196/66582 (PMC12349888; doi:10.2196/66582)
Supplement: Multimedia Appendix 2 [file formative-v9-e66582-s002.pdf]

Supplementary Table 2. Overview of Platform Architecture and Security Features

The platform is hosted on the University Laval web infrastructure, ensuring compliance with institutional IT policies. The architecture follows a client-server model with the following components:

- **Frontend**: Developed using modern web technologies (Blazor/React.js/Vue.js) for an intuitive user experience.
- **Backend**: Implemented using a secure framework (Dotnet/Node.js/Django/Laravel) to handle authentication, data processing, and API endpoints.
- **Database**: A relational database (SQL/PostgreSQL/MySQL) secured with encryption and access control measures.
- **Hosting**: Deployed on University Laval's managed servers, with routine security updates and maintenance.
- Summary Table of Security Features

**Security Feature**	**Description**
SSL Encryption	TLS 1.2+ for secure data transmission
Authentication	Password secured
Access Control	Role-Based Access Control (RBAC)
Data Encryption	AES-256 encryption for data at rest
Firewalls	University Laval's firewall protection
Backups	Automated encrypted backups
Logging	Activity monitoring
Compliance	Adherence to GDPR & institutional policies
